# What are the causes of non‐tolerance to new spectacles and how can they be avoided?

**DOI:** 10.1111/opo.12961

**Published:** 2022-02-14

**Authors:** Jeremy Beesley, Christopher J Davey, David B Elliott

**Affiliations:** ^1^ Bradford School of Optometry and Vision Science University of Bradford Bradford UK

**Keywords:** non‐tolerance, oblique cylinders, prescribing maxims, progressive addition lenses, recheck examinations

## Abstract

**Purpose:**

To investigate non‐tolerance cases from several UK practices to determine their likely causes and how they might have been avoided.

**Methods:**

Patient complaint and refraction data were collected from non‐tolerance recheck examinations. For one practice, clinical data were also collected retrospectively to investigate the quality of the eye examinations.

**Results:**

Data for 279 rechecks were gathered from 10 practices and a recheck frequency of 2.3% was found. The mean patient age was 60 (SD 16) years, with cylinder changes responsible for 38% of prescription‐related causes of rechecks, overplusing or underminusing 26%, and underplusing or overminusing just 11%. An assessment of 242 recheck corrections found that 40% were unsatisfactory (e.g., failed to address initial or recheck symptoms, *N* = 45) and retrospective analysis of 217 case records showed many limitations (e.g., 61% or 28% recorded no uncorrected or habitual visual acuity (VA) at either initial examination or recheck).

**Conclusions:**

Given that overplus‐underminus was a much bigger proportion of prescription‐related cases than overminus‐underplus (26% vs. 11%), the refraction mantra of “maximum plus for maximum VA” should be balanced by increased teaching of the problems of overplusing and underminusing, and the use of prescribing guidelines. In addition, continuing professional development regarding the basics of the recheck examination, refraction, visual acuity and prism determination is needed. Changes of oblique cylinders should be carefully considered in older patients as this is a common cause of non‐tolerance. In addition, if the “if it ain't broke, don't fix it” and related maxims had been applied to all patients who were asymptomatic at the original examination, one third of all non‐tolerance cases could have been avoided. Finally, it would seem appropriate for practices to develop a system to deal better with non‐tolerance cases. Perhaps an experienced clinician should examine all patients with non‐tolerance and provide feedback to the original clinician.

**Key points**

- Patient dissatisfaction with new spectacles following an eye examination was found in 2.3% of eye examinations conducted, which corresponds to more than 0.5 million rechecks per year in the UK.
- Eighty‐three percent of 279 rechecks were considered to be due to poor quality refractions, with minimal understanding shown of the importance of associating refractive correction change with symptoms and/or visual acuity.
- Refraction and prescribing needs substantially more emphasis in continuing education and training.

## INTRODUCTION

In a recent systematic review and meta‐analysis of spectacle non‐tolerance studies, Bist et al.^1^ reported a pooled prevalence of spectacle non‐tolerance of 2.1% of eye examinations. This indicates that approximately 473,340 (2.1% of 22.54 million^2^) rechecks are performed each year in the UK: nearly half a million unhappy patients returning to practices for further assessment, and in many cases, reglazed spectacles at no cost to the patient. These patients are also likely to be the tip of the iceberg, in that it is probable that many other patients do not return but are unhappy with their spectacles.^1^, ^3^ Research suggests that some dissatisfied patients will also broadcast their dissatisfaction by word of mouth to at least nine people, with 13% of dissatisfied customers telling more than 20 people^4^; this figure is likely an underestimation given the huge increase in communication via social media in recent years. Given the scale of this issue and its importance to the reputation of practices and the profession more generally, it is surprising that so little research has been performed in this area. Bist et al.^1^ reported data from only four studies, of which one was from a UK community practice.^5^ Bist et al. also highlighted limitations in these studies,^1^ including the heterogeneity in the data due to the small number of studies and the small sample sizes in some of those studies (e.g., non‐tolerance data from 53^3^ and 62 patients^5^ limiting interpretation, and that all studies assumed that patients not returning with complaints after the recheck examination were satisfied with their new spectacles). In this study, we attempted to overcome some of the limitations of the earlier studies and investigated a relatively large number of non‐tolerance cases (*N* = 279) from practices that were part of a large UK multiple practice, and for a proportion included a check‐up of their satisfaction with any new spectacles after the recheck examination. We tested the following hypotheses: (1) Patients older than 65 years suffer more with non‐tolerance issues;^6^ (2) Over‐plused/under‐minused corrections are more of a problem than over‐minused/under‐plused,^3^, ^5^, ^7^ despite clinicians being taught “maximum plus for maximum VA”;^8^, ^9^, ^10^, ^11^ (3) Oblique cylinder changes are particularly troublesome^12^, ^13^, ^14^, ^15^, ^16^, ^17^ and (4) Many non‐tolerances could be avoided if prescribing maxims such as “if it ain't broke, don't fix it”,^13^, ^14^, ^15^, ^17^, ^18^, ^19^ “if it ain't broke, don't fix it much”,^17^, ^19^ and “if it ain't broke don't fix it (distance specific)”^17^, ^19^ were used more.

## METHODS

Ethical approval was obtained from the University of Bradford Ethics Committee (EC24979). A pilot study in two Northern England optometry practices that were part of a large group of optical practices was used to develop a recheck summary form based on the ‘Concern Handling Form’ used at the time by those practices. It highlighted the problem of getting optometrists to complete the form and was therefore simplified. The form used in the main study is provided in Appendix 1. The form was signed by the patient to confirm consent to the use of their anonymised data. Part C was completed after a telephone call to the patient, 10 to 14 days following collection of remade spectacles. The determination regarding whether the patient was happy with the outcome of the recheck in practice A was made by a telephone call from an experienced dispensing optician in one practice (owned by author JB) who approached the enquiry from a customer service courtesy call perspective. Data were gathered from a small number of practices within a large group in Northern England, which ensured a consistent policy of dealing with patient complaints and consistent methods of data collection. On receipt of any complaint, dispensing staff investigate and attempt to remedy any lens or glazing issues; however, any further, prescription‐related problems would lead to a recheck examination being booked with an optometrist. There was no formal procedure regarding who the recheck optometrist should be. Recheck frequency data were gathered using practice reports which yielded the total number of examinations and rechecks performed, together with the conversion rate (i.e., the percentage of dispensed patients relative to the total examinations performed) to determine the number of dispenses from the examinations. This gave the recheck rate independent of the number of data forms gathered. The consistent type of reports across practices within the group was an important factor in the decision to confine the study to this one large group of optical practices.

Practices were recruited from personal contacts within the group and requests for participants posted on group forums. Approximately 25 practices initially indicated a willingness to participate, and forms were printed on green paper to make them stand out in a busy practice, and posted to the practices together with an explanation sheet regarding how to recruit subjects, follow the study guidelines and complete each form. The main method used for contacting participating practices was email.

### Data analysis

An assessment of the recheck examinations was made independently by two UK optometrists, with 28 (DE) and 23 (JB) years of experience, based on the information of the habitual correction and any symptoms, the non‐tolerated correction and any symptoms, and the recheck correction and any indication that the patient reported being ‘happy’ with the recheck correction. These assessments included a brief synopsis of each case plus the likely perceived cause(s) of the recheck and the quality of the recheck. Any initial differences of opinion were resolved by a subsequent review that included inspection of the other assessment, and in the case of disagreement, by author CD (15 years optometric experience). To test hypothesis 4 regarding the potential usefulness of the clinical maxims “if it ain't broke, don't fix it”,^13^, ^14^, ^15^, ^17^, ^18^, ^19^ “if it ain't broke, don't fix it much”^17^, ^19^ and “if it ain't broke don't fix it (distance specific),” all patients with no reported symptoms at one (i.e., distance or near) or both distances in the initial examination were assessed. We deemed that the use of the maxims would have avoided a non‐tolerance in cases where the patient complained of problems (distance and/or near blur, etc.) at the recheck examination, but was happy with the recheck correction (and the habitual correction assuming the initial report of no symptoms was correct). If the correction was returned exactly to the habitual refraction, then we deemed that the maxim “if it ain't broke don't fix it” would have avoided the non‐tolerance. Some patients might have a gradual change in prescription since their last eye examination which might not give symptoms, or perhaps a patient wearing progressive addition lenses (PALs) / varifocals might be asymptomatic due to a backward head tilt to compensate for an increase in hyperopia. Such patients would benefit from a small change, which if less than half the difference between the habitual and new prescription, we classified as an “if it ain't broke don't fix it much” case. An “if it ain't broke don't fix it (distance specific)” case typically described a situation where the patient was happy with their distance vision at the initial examination (but was complaining of near blur) and returned for a recheck to indicate that a change in the distance correction had led to distance vision blur.

In one practice (A) and on rare occasions, a dispensing optician (DO) had made a binocular change to the reading addition power in response to a specific patient complaint of an incorrect near working distance, where no distance vision problems were reported. The Association of British Dispensing Opticians (ABDO) indicate that changes in prescription to adjust for back vertex distance and near working distance requirements such as these are permissible for UK‐trained DOs,^20^, ^21^ and optometric colleagues were available for consultation if required. However, it should be noted that such adjustments might not be permissible in all countries. These rechecks fell outside the standard recheck process and to remedy this, collection of these data started in March 2021.

Statistical analyses were performed using SPSS v26 (IBM, ibm.com). The distribution of data was assessed using kurtosis and skewness assessments, and data were described using mean and standard deviation (SD) for normal distributions or median and inter‐quartile range (IQR) for non‐normal distributions. To investigate and compare frequencies of eye examinations and recheck examinations, linear regression analyses were conducted for eye examinations and recheck examinations (dependent variables) versus patient age (independent variable). To investigate whether older groups complained of symptoms sooner than younger groups, a linear regression analysis was also conducted for the time to complaint (dependent variable) versus patient age (independent variable).

Sphero‐cylindrical prescription data were converted to power vectors^22^ for calculation of means and rounded to 0.125 D when converted back to sphero‐cylinders.

## RESULTS

Recheck forms were received from 10 practices (Table 1). Some practices contributed very few forms and for just a short period and others stopped data collection after eight months, leaving just author JB’s practice A to continue for a full 20 months. In practice A, over the 6‐month period March to August 2021, six patients had their reading addition changed by a DO to adjust the working distance. No other participating practice made such changes, with all their prescription‐related problems booked for an optometrist recheck.

**TABLE 1:** Recheck frequency (May 1, 2018 to December 31, 2019)

Practice	Rechecks, *N* (% of dispensed spectacles)	Rechecks (% of eye examinations)	Period of data collection	Forms received. *N* (% of 261 optometrist rechecks)
A	471 (3.0%)^a^	(1.7%)^a^	20 months	199 (41%)
B	224 (4.9%)	(2.9%)	8 months	24 (11%)
C	321 (6.0%)	(3.5%)	8 months	10 (3%)
D	129 (4.9%)	(3.2%)	8 months	6 (5%)
E	250 (5.4%)	(3.6%)	8 months	4 (2%)
F	69 (1.8%)	(1.2%)	4 months	3 (4%)
G	14 (1.2%)	(0.7%)	2 months	2 (14%)
H	87 (4.3%)	(2.7%)	2 months	2 (2%)
J	63 (2.7%)	(1.7%)	4 months	0
K	93 (0.8%)^b^	(0.6%)^b^	4 months	11 (12%)
Overall	1628 (3.9%)	1628 (2.3%)		261 (15%)

^a^ Includes 18 dispensing optician (DO) reading addition adjustments for the 1.5 year period of the main study, calculated from the 6 over the 6‐month period that these data were collected.

^b^ The very low 0.8% figure was deemed unreliable due to the way optometrists were remunerated and thus these values were discarded in the overall frequency figures.

A total of 261 recheck forms were returned, with a mean patient age of 60 years (SD 16 years). Patient age had skewness of −0.81 and kurtosis 0.27, suggesting an approximately normal distribution. The median number of days to complaint was 7 (IQR 2–23), with skewness of 2.07 and kurtosis 4.15 suggesting a skewed distribution. A linear regression investigating any relationship between the patient age and time (days) to complaint showed no significant correlation (*p* = 0.51).

**FIGURE 1:**
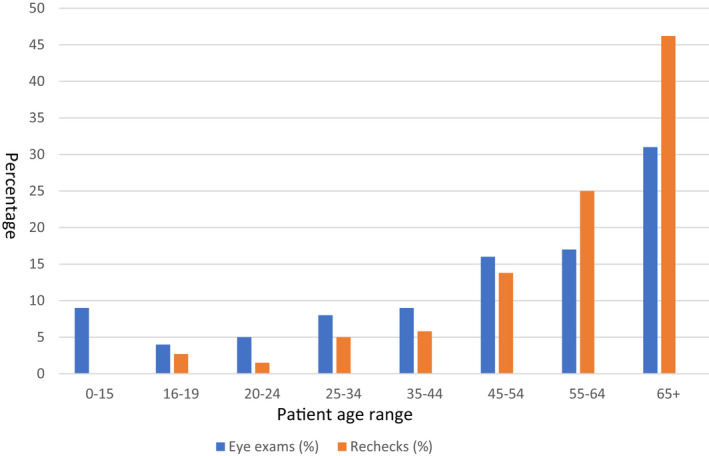
Percentages of eye examinations and rechecks as a function of patient age in years

Figure 1 investigates whether this distribution of rechecks could be due to the age distribution of all eye examinations conducted, and so shows the relative number of eye examinations and rechecks. Although eye examination percentages increased at 4.5% per decade (*p* = 0.003, 95% CI. 2.3–6.6), rechecks increased at the higher rate of 7.3% per decade (*p* = 0.01, 95% CI. 2.7–11.9). To allow a fair comparison given that presbyopic patients could complain of near and intermediate problems (due to an incorrect near addition) as well as distance problems, a linear regression was repeated excluding presbyopic patients with exclusively near and intermediate problems: distance correction rechecks increased at 6.8% per decade (*p* = 0.01, 95% CI. 2.4–11.1).

Table 2 lists the most common recheck complaints reported by patients, as recorded by the rechecking optometrist.

**TABLE 2:** Principal symptoms recorded at recheck appointments (*N* = 279, including dispensing optician (DO) reading addition adjustments). A total of 101 (36%) recorded more than one symptom

Symptom	*N* (%), Total > 100%	*N* who reported just one symptom
Blurred distance vision	123 (44%)	76
Blurred near vision	75 (27%)	38
Working distance too close	30 (11%)	24
Diplopia at distance or near	26 (9%)	15
Spectacles too strong at distance or near	25 (9%)	12
Strain at distance or near	24 (9%)	11
Distortion	14 (5%)	4
Dizziness	13 (5%)	2
All others	<10 (<4%) each.	

### Assessment of cases

An error in refractive error measurement was deemed to be responsible for 200 of the 279 rechecks (71.7%) with a failure to adapt to an accurate prescription deemed responsible for the remaining 42 (15.1%) of the prescription‐related rechecks. Lens type issues accounted for 5.7% (*N* = 16), pathology 5.0% (*N* = 14) and data input errors 2.5% (*N* = 7). The data input errors were mainly caused by a digit being missed off the sphere, cylinder or axis (five cases), a digit duplicated (one case, “55” typed instead of “5”) and one case where a “+” was entered (the default computer sign) instead of a minus. A categorisation of the likely causes of the 242 prescription‐related rechecks in terms of the type of refractive change is provided in Table 3. There were similar percentages of rechecks with oblique cylinder changes (30 to 60 degrees and 120 to 150 degrees) compared to with‐ and against‐the‐rule changes: both 14 (50%) in those patients who failed to adapt and 49 (40%) oblique cylinder changes versus 73 (60%) non‐oblique changes where there was a deemed error in refractive correction.

**TABLE 3:** Categorisation of the likely causes of the 242 prescription‐related cases: 54% had one likely cause, 41% had two and 5% had more than three causes. Rx: spectacle prescription

Causes of the complaint	Error of Rx Patient *N* = 200 (% of total 302 causes)	Fail to adapt to accurate Rx Patient *N* = 42 (% of total 54 causes)
Over‐plused	58 (19%)	3 (6%)
Under‐minused	28 (9%)	3 (6%)
Under‐plused	14 (5%)	5 (9%)
Over‐minused	19 (6%)	2 (4%)
Cylinder changes	109 (36%)	25 (46%)
Add too high	27 (9%)^a^	3 (6%)
Add too low	11 (4%)	2 (4%)
Anisometropia change	22 (7%)	9 (17%)
Prism	14 (5%)	2 (4%)
Total	302 (100%)	54 (100%)

^a^ Includes nine changes to the reading addition made by the rechecking optometrist in the main study's 18‐month data collection period, plus 18 changes made by a dispensing optician, calculated from the 6 made in a separate 6‐month data collection period.

Of the 242 prescription‐related cases, the recheck prescription differed from the non‐tolerated prescription by +0.50 D or less in 209 (86.4%) cases.

### Lens type issues

Sixteen (6%) rechecks involved a change of lens type that was implicated in the cause of the non‐tolerance. Of note is that PALs were involved in 13 of the 16 (5 first‐time PAL wearers, 7 previous PAL wearers and 1 previous bifocal wearer), with the other changes being two base curve changes and one pair of intermediate single vision spectacles that needed to be dispensed specifically for computer use.

PAL wearers were involved in 111 of 276 rechecks (40%), bifocals in 38 (14%) and single vision lenses in 127 (46%). Presbyopic single vision lens wearers were involved in 88 (32%) rechecks. Across a 12‐month period for practice A, 24% of all dispenses were PALs, 6% bifocals and 70% single vision.

### Patients reporting blurred distance vision at the recheck

This was the most common reported complaint, at 44% of all rechecks (Table 2). The rechecked patients who reported symptoms of blurred distance vision were considered in two groups: those over 60 years of age who might confidently be expected to have little or no accommodation (*N* = 67), and those under 50 years who might confidently be expected to have some accommodation (*N* = 29).^23^ The 29 patients aged 50 to 60 years are not considered here. The mean change from the habitual to the initial rechecked spectacle correction was calculated using absolute values^22^ and rounded to 0.125 D for the two refractive change types with a reasonable sample size. The mean over‐plus/under‐minus was 0.50 DS in both age groups and the mean cylinder change was 0.375 DC in both age groups.

### Oblique cylinders

Of 279 rechecks, 134 (48%) were assessed as having cylinder changes likely to have contributed to the non‐tolerance. Of these, 63 (23% of all rechecks) were oblique changes and 87 (31%) were either with‐the‐rule or against‐the‐rule changes. Some patients had an oblique cylinder in one eye and with‐ or against‐the‐rule astigmatism in the other. Of the 63 patients for whom oblique cylinder changes were assessed as contributing to the symptoms, 13 (21%) reported symptoms of dizziness, nausea or distortion (including sloping floor or edges).

Considering this range of symptoms reported at the recheck (dizziness, nausea and distortion) and the 34 patients (12% of the 279 rechecks) who reported these symptoms, 13/34 (38%) had oblique cylinder changes, 13/34 (38%) had either with‐ or against‐the‐rule changes and 8/34 (24%) did not have cylinder‐related changes in prescription; these were deemed to be due to over/under‐plus/minus, prism and changes in anisometropia. Of the 66 patients reporting a problem within two days, 46 (70%) had cylinder changes, of which 23 (35%) were oblique axes.

### Quality of rechecks

Of the 242 prescription‐related optometrist rechecks, 146 (60%) were assessed as satisfactory and 96 (40%) unsatisfactory. Reasons for the assessment being designated as unsatisfactory included issuing a prescription which failed to address the problem of either the initial examination or the recheck (*N* = 47); one problem was fixed but likely induced another (*N* = 26; e.g., remedying an under‐minus issue but prescribing vertical prism when no binocular vision issue was indicated); no change in prescription found but where a change appears to have been required from the symptoms (*N* = 11); a need for a 2nd‐recheck (*N* = 8) or a refund was required (*N* = 4). All 15 patients (100%) that reported being unhappy at the 2‐week post collection follow‐up phone call were independently assessed as having received an unsatisfactory recheck examination. However, 29 patients were deemed to have received an unsatisfactory recheck examination, but reported being happy at follow‐up. Of the six reading addition changes by the dispensing optician, three (50%) were deemed unsatisfactory in that they were unlikely to fix the problem.

Due to the large number of recheck examinations that were deemed unsatisfactory, a further, retrospective assessment of the case records of the 217 rechecks of practice A was conducted. The measurement (or recording) of habitual visual acuity (VA) was rare and just 24 (11%) recorded habitual monocular VA at the time of the original examination, 22 (11%) had either vision or habitual VA but only taken at the recheck, and 44 (20%) had only binocular habitual visual acuity recordings. Sixty‐one (28%) recorded no visions (i.e., unaided VA) or habitual VA at either the first examination or recheck. There were 18 cases where prism was assessed as the primary problem. Of these, 21 of the 36 first examinations or rechecks had a cover test as the sole binocular vision test, with no fixation disparity, fusional reserves, accommodation or convergence test recorded. Of these 21, three had “no movement” recorded in contradiction to a prior or subsequent cover test (which recorded, for example, “large exophoria”), thus bringing doubt upon that test. There was one test and five rechecks in which prism was the primary problem where no binocular vision test was recorded. Four cases had prism prescribed at the recheck but with no prism in the habitual prescription, no symptoms suggesting any binocular vision issue and no binocular vision test conducted (Table 4).

**TABLE 4:** Errors in refractive correction causing rechecks in patients reporting blurred distance vision. Some patients were assessed as having multiple causes, so percentages sum to greater than 100

	Patients without accommodation (>60 years), *N* = 67	Patients with some accommodation (<50 years), *N* = 29
Over‐plus / under‐minus	31 (46%)	17 (59%)
Cylinder changes (axis or power)	40 (60%)	12 (41%)
Under‐plus / over‐minus	3 (4%)	3 (10%)
Other causes (e.g., anisometropia, pathology, balance issues)	11 (16%)	4 (14%)

There were 56 patients with a large prescription change as defined by Cumming et al.,^6^ of which, 21 (38%) records showed that a warning had been given at the time of the examination, while a further 16 (29%) recorded such a warning at the recheck. Nineteen (34%) of cases had no record of a warning at either first examination or recheck (Table 5).

**TABLE 5:** Outcomes of a follow‐up telephone call. *N* = 161 (58% of all rechecks)

Happy	146 (91% of those contacted)
2nd‐recheck booked	8 (5%)
Refunded	4 (2%)
Further lens advice required	2 (1%)
Original prescription reglazed	1 (<1%)

### Would the application of maxims have avoided some non‐tolerance cases?

Table 6 summarises the 96 cases (34.4% of rechecks) where the patient reported no symptoms with their habitual prescription at the time of the initial examination and the 17 where only near problems were reported at the initial examination but distance problems were reported at the recheck. A prescription was deemed as having been returned to the habitual finding if the sphere and cylinder powers were exact and the cylinder axis within just a few degrees (axis change of ≤10 degrees for cylinder <0.75 DC and ≤5 degrees for cylinder ≥0.75 DC).

**TABLE 6:** Recheck outcome of cases where the patient reported no problems at the initial examination (*N* = 96) and no distance vision problem at the initial examination but distance problems were reported at the recheck (*N* = 17)

Recheck outcome	*N* (%). Total = 100%	Patient reported being happy with the recheck result (% of those contacted.)	Patient not happy^a^ (% of those contacted.)	Patient not contacted
Returned to habitual Rx.	14 (12%)	7 (100%)	0	7
Returned to within ½ the difference, Test‐Habitual	41 (36%)	23 (96%)	1 (4%)	17
More than ½ the difference, Test‐Habitual, maintained	58 (51%)	30 (81%)	7 (19%)	21

^a^ Patient designated as “not happy” if the recheck resulted in a further recheck, a refund, or the original prescription was reglazed.

A specific cause of a recheck was where the patient reported problems with their near vision only, with no distance problems, but the distance prescription was subsequently changed with ensuing distance vision problems causing a recheck. There were 17 (6.1% of 279 rechecks) such cases and of these 17, three were returned to the habitual prescription, two were returned to within half the difference of test to habitual and 12 were returned to more than half this difference. These data are included in Table 6.

## DISCUSSION

Patients reporting problems with new spectacles experience a cost in time to themselves and cause financial and reputational costs to the practice. While the numbers of such patients are a small percentage of all patients seen in practice, they represent large numbers of dissatisfied patients across the optical industry. Surprisingly, there are few studies^1^ that investigate the causes of dissatisfaction. In this study we aimed to investigate the causes and offer suggestions for how these numbers might be reduced.

Various causes were implicated in patient dissatisfaction: over‐plusing/under‐minusing was more than twice as likely to cause problems than under‐plusing/over‐minusing; cylinder errors were the most common cause of dissatisfaction and with oblique cylinder changes particularly implicated; presenting symptoms are often not being reconciled with changes in prescription and improvements in visual acuity and while practitioners may consider reports of dissatisfaction as “non‐tolerance”, an error in the determination of the prescription was nearly five times more common than a non‐tolerance to a prescription change. Reconciling presenting symptoms with prescription changes and improvements in acuity are particularly important for the asymptomatic patient, for whom application of a maxim, “if it ain't broke don't fix it (much),” would have saved one third of all rechecks.

### Recheck frequency

The overall recheck frequency as a proportion of eye examinations conducted (prescriptions written) was 2.3%, which is similar to the earlier smaller sample of UK high street practices^5^ at 2%, and the pooled frequency of recheck studies of 2.1%.^1^ As it is the spectacles, and not an undispensed prescription, which cause the patient to report problems, it is perhaps more relevant to quote the recheck frequency as a proportion of spectacles dispensed, which was 3.9%. Just one previous optometric study quoted both figures: 3% of eye examinations and 5.7% of spectacles dispensed.^3^ Although these percentages appear small, they correspond to large numbers of patients who are dissatisfied with their spectacles following an eye examination, with cost and reputational implications for the practice.

### Patient age

The mean age of rechecked patients at 60 years is similar to previous studies of 50 to 60 years of age.^5^, ^19^, ^24^, ^25^ The age increase in distance correction rechecks of 6.8% per decade, compared with the increase in numbers of examinations at 4.5% per decade, suggests that older people have more problems leading to a recheck. This seems likely to be due to older patients having greater difficulty adapting to new spectacles,^6^, ^26^ but more research could consider alternative aetiologies, such as whether patient concerns regarding new spectacles and confidence in the optometrist change with age. This study was based on practitioner‐reported factors; however further insight would likely be gained through PROMs (Patient Reported Outcome Measures).^27^

### Clinician error versus failure to adapt

Of the prescription‐related errors, inaccurate measurements of refractive error were nearly five times greater than failures to adapt to an accurate prescription. Freeman and Evans^5^ found identical figures of prescription‐related causes of rechecks of 83% (*N* = 30) and 17% (*N* = 6), respectively, although their assessment was made by the rechecking optometrist rather than a retrospective assessment, as in this study. While some patients may be expected to struggle to adapt to a new spectacle prescription, this illustrates that the significant majority of rechecks are caused by optometrist “error”. However, input errors were relatively low, at 2.5% of all rechecks compared to an average figure from the literature of 8.7%,^1^ and this suggests that useful checking processes were being used.

### Lens type

Multifocal (PAL and bifocal) wearers accounted for 54% of rechecks compared with 30% of dispenses; however as older age groups are more likely to report problems (Figure 1), it is difficult to draw conclusions whether some lens types increase the likelihood of reporting problems. Of the 16 lens‐type issues causing a recheck, 13 were PALs, although with similar proportions of rechecks versus dispenses for PALs and bifocals, PAL wearers appear no more likely to report problems than bifocal wearers.

### Multiple causes of rechecks

Hrynchak^7^ and Freeman and Evans^5^ determined the principal cause for each recheck, although the latter found 7% (*N* = 2) had both sphere and cylinder errors. In many of the 242 prescription‐related cases of this study it was unclear which could be regarded as the principal cause, and consequently, multiple causes were permitted, with two or more determined for 46% of the 242 prescription‐related cases. The requirement for allowing multiple causes is most clearly demonstrated with one 57‐year‐old, who reported problems following significant increases in their hyperopic correction and oblique cylinder powers, had prism changed and was then dispensed a PAL for the first time. Indeed, we propose that making multiple changes to a refractive correction, particularly in older patients, makes it more likely that this will lead to a recheck.

### Over‐plus and under‐minus versus under‐plus and over‐minus

Over‐plusing or under‐minusing was found to be more than twice as likely as over‐minusing or under‐plusing (28% vs. 11% of causes), with very similar figures to previous studies of 26%^7^ and 28%.^5^ This was even more dramatically demonstrated in patients complaining of distance vision blur at the recheck examination (Table 4), where 50% were over‐plused or under‐minused, and a mere 6% over‐minused/ under‐plused. The greater likelihood of over‐plusing or under‐minusing patients of all ages suggests that the refraction mantra of “maximum plus for maximum VA”^8^, ^9^, ^10^, ^11^ is overwhelming any teaching of the prescribing problems that this mantra can lead to.

The average prescription changes for those reporting blurred distance vision were found to be roughly equal at ±0.50 DS and ±0.375 DC. Considering all 242 prescription‐related cases, 86% had a recheck prescription differing from the non‐tolerated prescription by ±0.50 D or less. This correlated well with the findings of Freeman and Evans,^5^ who found 84% of the prescription changes with their rechecks were within ±0.50 D. These figures highlight that most rechecks are due to relatively small dioptric differences.

### Non‐tolerance of cylinder changes

The greatest proportion of the 302 prescription errors was found to be cylinder power and/or axis errors, at 36% (*N* = 109). These are higher than previous findings of 26%^7^ and 27%.^5^ However, of the 134 errors of prescription or failure to adapt cases where cylinder changes were implicated, just 25% (*N* = 33) had this as the sole cause, so considering multiple causes in this study likely explains some of the difference to the earlier investigations.^5^, ^7^ Differences to previous findings may also be related to the use of autorefractors rather than retinoscopy and/or perhaps a partial prescribing approach to cylinder changes in those studies. All participating practices here used an autorefractor to measure the objective prescription and most practitioners are likely to have used this result rather than performing retinoscopy. Modern autorefractors show good agreement with subjective results,^28^ particularly in astigmatism measurement, and perhaps practitioners accepted the autorefractor result as an “accurate” finding. If so, this might lead to over‐correcting if the practitioner does not consider partial prescribing to aid patient adaptation.

### Oblique cylinders

Only about 20% of astigmatism is oblique^29^ so that oblique axis changes would be expected to produce 20% of rechecks. However, rechecks deemed to have been due to oblique axes were found in 42% of astigmatic change cases (*N* = 63) so that patients with oblique axes changes were about twice as likely to report problems (Fisher's exact test, *p* < 0.0001). In particular, non‐tolerance patients with oblique cylinder changes complained of dizziness‐related symptoms (13/63, 21%), and of 30 cases who did indeed report dizziness‐related symptoms, 13 (43%) had oblique cylinder changes, which supports reports of increased dizziness with oblique cylinder changes following cataract surgery.^16^ Of the 66 patients reporting a problem within two days, 46 (70%) had cylinder changes (23 oblique changes, 35%), suggesting increased unacceptability of these types of change.

### Quality of tests, rechecks and records

The overall quality of measuring and recording habitual VAs was found to be poor, with 28% of cases having neither unaided nor habitual VA recorded at either the first examination or recheck. In the rechecks, where the practitioner might be considered as being under particular pressure to remedy the patient's problems, just 11% had either unaided or habitual acuities recorded. As 20% of cases had only binocular habitual VA recorded, nearly half of cases had insufficient habitual VA data to assess the benefits of prescription changes. To avoid the risk of over‐plusing, under‐minusing and over‐correcting cylinder powers it seems logical to only change a prescription as far as improvements in acuity can be made; however, poor recording of acuities renders this impossible. Assessments of binocular status were also poor, with a lack of assessment of tests to help determine the need for and magnitude of any prism.

Nearly half of the rechecks were assessed as unsatisfactory because the practitioner issued a prescription that failed to address the symptoms leading to a recheck and/or the symptoms in the initial examination. This suggests that the optometrists involved were treating the refraction in isolation and were not reconciling prescription changes with symptoms. This apparent lack of reconciling symptoms with changes in prescription, improvements in acuity and binocular tests brings into question the approaches optometrists are taking: is the subjective refraction prescribed without consideration of any other clinical information? Of the six changes in near addition performed by the dispensing optician, three were deemed unsatisfactory as they would likely not have fixed the problem, which was due to changes in the initial distance correction. It seems appropriate that all patient prescription‐related complaints should be referred to an optometrist, even if the problem appears to be simply a near working distance issue.

An integral part of any eye examination should be the explanation of results and managing patient expectations, with the provision of more information reducing the number of patients who are dissatisfied with new spectacles.^26^ The records of 38% of patients with large prescription changes had a documented warning of adaptation at the time of the initial examination. This represents an improvement from the 8% of practitioners reporting discussing patients’ expectations with new spectacles in 2005.^26^

### Following up rechecked patients

Of the 161 patients followed up after collection of the remade spectacles, 146 (91%) reported being happy with the outcome. An unscripted ‘courtesy call’ approach was used to reduce the burden on the patient and staff member compared with a more scripted interview or survey and this likely limited its accuracy. Indeed, the high proportion of happy patients is somewhat surprising given that 40% of rechecks were assessed as unsatisfactory. Following up patients who have collected remade spectacles after a recheck should be seen by practices as a positive policy, as the majority of patients are likely to be happy, will view this as good patient care and it presents a further opportunity to remedy any complaint that may persist.

### Prescribing maxims of “if it ain't broke...”

In approximately 50% of the rechecks of asymptomatic patients, more than half the difference between the habitual and initial examination prescriptions was maintained, despite the patient having no reported problems with their habitual prescription. The recheck correction was thus closer to the initial correction (that was the cause of the non‐tolerance and led to symptoms) than the habitual correction that caused no symptoms. This suggests that the optometrists were not thinking about the link between symptoms and prescription changes when prescribing. The clinicians appeared to just prescribe the subjective refraction result, with some cylinder values seemingly dependent on autorefractor results. Seven of 37 (19%) of those contacted reported being unhappy with the recheck result, and although it is perhaps surprising that this wasn't higher, relatively few reported being unhappy with the recheck spectacles (15/96), and so these seven represent nearly half the unhappy recheck patients. When the correction was returned to the habitual (7) or less than half the difference between the habitual and initial correction (24), virtually all (7 and 23 respectively; Table 6) of those contacted reported being happy with the recheck correction. Considering the 279 cases of the whole study, this suggests that if one of the maxims had been applied at the initial examination, one third of all rechecks could have been avoided.

A particular type of distance‐specific “if it ain't broke don't fix it” patient was seen (*N* = 17, 6% of all rechecks) in which the patient reported near vision problems but none at distance, and yet the initial correction resulted in distance vision problems following a change to the distance prescription. If the clinicians had applied the maxims ““if it ain't broke don't fix it” or “if it ain't broke don't fix it much” to the distance correction and then altered the near addition appropriately to correct the near vision symptoms, these non‐tolerance cases could also have been avoided. However, that is not to say that a sole complaint of reduced near vision should only be remedied with an adjustment to the near addition. Even if the patient reports being happy with their current spectacles for distance vision, it is possible they haven't noticed a subtle prescription change from which they may benefit, or they are compensating for a small hyperopic increase or reduction in their myopia with a backward head tilt with PALs. Consider a 55‐year‐old patient, +1.00DS R and L, with a +2.00D Add reporting problems reading and a full subjective refraction result of +1.50DS R and L, +2.00D Add. Applying a strict, “if it ain't broke don't fix it”, +1.00DS R and L with a +2.50D Add would fix the reported near problem. However, a better solution might be to apply the “if it ain't broke don't fix it much” maxim and prescribe +1.25DS R and L with a +2.25 Add. Any improvement in distance VA from the habitual VA would particularly suggest this approach. This would have the benefit of giving extra help for mid‐distances and not giving such a steep gradient of prescription change with the PALs, potentially easing adaptation.

### Limitations of the study

Data for this study were gathered from 10 practices of one large group, with 41% of all recheck forms derived from one practice. It might therefore be questioned whether these results are generalisable to UK optometry. The recheck rate (as a proportion of prescriptions written) for practice A of 1.7%, compared with 2.3% of the whole study, 2.0% for the earlier UK high‐street practice study^5^ and 2.1% from a meta‐analysis of previous studies,^1^ indicates that recheck rates from practice A are at least as good as the profession as a whole.

## CONCLUSIONS

The recheck frequency of a small number of practices within a large group was 2.3% of eye examinations (3.9% of dispensed patients) with a mean age of 60 years (SD 16 years). An inaccurate measurement of refractive error accounted for 83% of prescription‐related rechecks and a failure of patient adaptation to an accurate prescription for just 17%. When this is coupled with very limited measurement of monocular habitual VA, there is a need for more emphasis on refraction in optometrist continuing professional development, and particularly the requirement for accurate VA, so that changes in prescription can be reconciled with improvements in acuity.

Changes in either cylinder power or axis accounted for 38% of all prescription‐related causes of rechecks, and patients with changes in oblique astigmatism were approximately twice as likely to report problems than those who have with‐ or against‐the‐rule astigmatism. The relatively greater problems provided by oblique cylinders needs to be emphasised in optometric education and continuing education. Over‐plusing or under‐minusing accounted for 26% of causes, while under‐plusing and over‐minusing accounted for only 11% of causes, so that the teaching mantra in subjective refraction of “maximum plus for maximum VA” needs to be balanced with greater teaching of prescribing maxims such as “cut the plus”, “don't reduce a happy myope” and “if it ain't broke don't fix it (much)”.^13^, ^14^, ^15^, ^17^, ^18^, ^19^

Patients who reported no problems at the initial examination accounted for more than one third of all rechecks. Where the recheck prescription was either returned to the habitual finding, or within half the difference of test‐habitual prescription, just one patient (4%) subsequently reported being unhappy. Where more than half this difference was maintained, 19% reported being unhappy. If either, “if it ain't broke, don't fix it”, or, “if it ain't broke, don't fix it much,” had been applied to such patients, one third of all rechecks could have been avoided. This needs great exposure in the optometry curriculum and continuing education.

Of the patients contacted after collection of their remade spectacles following a recheck, 91% reported being happy. To enhance customer care for the majority of patients who are likely to be happy and to remedy persisting problems of those who are not, it is recommended that practices contact patients after a recheck.

Finally, participating practices in this study had no formal processes regarding who should conduct rechecks. Given that 40% of recheck refractions were deemed unsatisfactory and few included any assessment of habitual VA or clinical tests to determine the need for and size of prism, rechecks could perhaps be improved if an experienced clinician who fully understands the links between habitual and subjective refraction, VA, symptoms and prescription changes, conducted all rechecks and provided the results and feedback to the original clinician.

## CONFLICT OF INTEREST

None.

## AUTHOR CONTRIBUTIONS

**Jeremy Beesley:** Conceptualization (equal); Data curation (lead); Formal analysis (lead); Investigation (lead); Methodology (lead); Project administration (lead); Resources (lead); Writing – original draft (lead); Writing – review & editing (lead). **Christopher James Davey:** Conceptualization (supporting); Data curation (supporting); Formal analysis (supporting); Investigation (supporting); Methodology (supporting); Project administration (supporting); Supervision (supporting); Validation (supporting); Visualization (supporting); Writing – original draft (supporting); Writing – review & editing (supporting). **David B Elliott:** Conceptualization (equal); Data curation (supporting); Formal analysis (supporting); Investigation (supporting); Methodology (supporting); Project administration (supporting); Supervision (equal); Validation (supporting); Visualization (supporting); Writing – original draft (supporting); Writing – review & editing (supporting).
